# 3D *Ex vivo* tissue platforms to investigate the early phases of influenza a virus- and SARS-CoV-2-induced respiratory diseases

**DOI:** 10.1080/22221751.2022.2117101

**Published:** 2022-09-21

**Authors:** Sebastian Schloer, Daniel Treuherz, Aileen Faist, Marlous de Witt, Katharina Wunderlich, Rainer Wiewrodt, Karsten Wiebe, Peter Barth, Joo-Hee Wälzlein, Susann Kummer, Anne Balkema-Buschmann, Stephan Ludwig, Linda Brunotte, Ursula Rescher

**Affiliations:** a Institute-Associated Research Group “Regulatory Mechanisms of Inflammation”, Institute of Medical Biochemistry, Center for Molecular Biology of Inflammation, and “Cells in Motion” Interfaculty Center, University of Münster, Münster, Germany; b Leibniz Institute of Virology, Hamburg, Germany; c Institute of Virology, Center for Molecular Biology of Inflammation, and “Cells in Motion” Interfaculty Center, University of Münster, Münster, Germany; d Department of Medicine A, Hematology, Oncology and Respiratory Medicine, University Hospital Münster, Münster, Germany; e Department of Thoracic Surgery, University Hospital Münster, Münster, Germany; f Gerhard-Domagk-Institute of Pathology, Westfälische Wilhelms-University, Münster, Germany; g Center for Biological Threats and Special Pathogens, Robert Koch-Institute, Berlin, Germany; h Friedrich-Loeffler-Institute, Institute of Novel and Emerging Infectious Diseases, Greifswald, Germany

**Keywords:** *Ex vivo* infection model, influenza, SARS-CoV-2, drug repurposing, innate immunity

## Abstract

Pandemic outbreaks of viruses such as influenza virus or SARS-CoV-2 are associated with high morbidity and mortality and thus pose a massive threat to global health and economics. Physiologically relevant models are needed to study the viral life cycle, describe the pathophysiological consequences of viral infection, and explore possible drug targets and treatment options. While simple cell culture-based models do not reflect the tissue environment and systemic responses, animal models are linked with huge direct and indirect costs and ethical questions. *Ex vivo* platforms based on tissue explants have been introduced as suitable platforms to bridge the gap between cell culture and animal models. We established a murine lung tissue explant platform for two respiratory viruses, influenza A virus (IAV) and SARS-CoV-2. We observed efficient viral replication, associated with the release of inflammatory cytokines and the induction of an antiviral interferon response, comparable to *ex vivo* infection in human lung explants. Endolysosomal entry could be confirmed as a potential host target for pharmacological intervention, and the potential repurposing potentials of fluoxetine and interferons for host-directed therapy previously seen *in vitro* could be recapitulated in the *ex vivo* model.

## Introduction

Influenza A virus (IAV) and SARS-CoV-2 are zoonotic viruses that primarily target the lung epithelium with a high risk of human-to-human transmission. Their outbreaks pose an enormous global burden both economically and on public health, therefore effective therapeutic and intervention strategies are urgently required to limit transmission and alleviate pathophysiology. Thus, preclinical *in vitro* models that adequately reflect the viral infection pathways, replication kinetics, and virus-induced cell damage are needed, to understand virus propagation in the relevant tissue environment and to provide more realistic test systems for drug screening. Widely used cell culture infection models utilise cultured airway-derived primary cells [1,2] or selected permissive cell lines such as the Calu-3 cell line (originally derived from human lung adenocarcinoma), that support efficient influenza A virus and SARS-CoV2 replication [3]. These models, which typically use monolayers of a single cell type have been successfully used to assess virus-host cell interactions [4], the kinetics of virus replication [5], cell-autonomous defense mechanisms [6], and to evaluate the efficacy of pharmaceutical interventions [7,8].

However, due to their lack of cell-type diversity, these relatively simple lung cell culture models do not provide insight into the complex pathophysiological processes in the target organ or even on a systemic level. In turn, the availability of more complex susceptible animal models is often limited, as has been observed with SARS-CoV-2. Since many SARS-CoV-2 strains do not readily bind to murine angiotensin-converting enzyme 2 (mACE2), laboratory mouse strains are either hardly infected or not at all [9], consequently requiring either the use of transgenic mice expressing human ACE2, or genetically modified virus strains [10]. Ferrets are susceptible to influenza virus and SARS-CoV-2 infection [11], and the golden Syrian hamster has been identified as a useful *in vivo* model to study SARS-CoV-2 transmission and pathogenesis [12,13]. In any case, such experimental approaches require specialised facilities, trained staff, and are cost-intensive. Moreover, the use of animals in scientific research is morally controversial. These ethical considerations have led to an increasing implementation of the 3R (Replace, Reduce, Refine) principle stating that animal experimentation is justified only if the expected benefit is high and cannot be achieved by available alternative methods. Further, the 3R principles requires the number of animals and their imposed harm to be kept to an absolute minimum, but sufficiently high to still allow experimental validity. To bridge the gap between cell culture and *in vivo* models, *ex vivo* infection models utilising tissue explants have been introduced as a suitable platform to study complex tissue-dependent mechanisms in infection, antiviral responses, and drug research [14]. *Ex vivo* infection of human tissues with relevant human respiratory pathogens, including seasonal and highly pathogenic avian influenza viruses (HPAIV) as well as SARS-CoV-2 was demonstrated and provided unique insights into the natural responses in humans [15–17]. However, the availability of human lung tissue is often limited and underlies ethical regulations. Therefore, the application of suitable animal-derived models is highly attractive.

To study pulmonary infections caused by enveloped RNA viruses in a murine model that more closely resembles the complex physiological lung environment compared to cell culture, we explored the potential of murine lung tissue explants as an *ex vivo* model for infection with the two respiratory viruses IAV and SARS-CoV-2. Both are enveloped viruses with a host-derived lipid membrane that harbours several viral membrane proteins required for successful virus entry. For the infection experiments, we used the mouse-adapted strains PR8 and the avian like strain SC35M, and the SARS-CoV-2 strain β-B1.351 that carries a spike protein mutation allowing murine infections [18]. Our data reveal that the C57BL/6-derived lung tissue supports efficient replication of both viruses, the mouse-pathogenic influenza virus SC35M and the SARS-CoV-2 strain β-B1.351, arguing that the *ex vivo* model is suitable for studying host innate immune responses to virus infection. Using this platform, we could confirm the relevance of endolysosomes as potential host cell drug targets during infection with these enveloped viruses, and we could recapitulate the antiviral activity of the virus-directed drug remdesivir and the potential repurposing uses of the host-directed drugs fluoxetine and interferons.

## Materials and methods

### Animals

C57BL/6 mice (Envigo, Huntingdon, UK) were kept in standard individually ventilated cages under specific pathogen-free (SPF) conditions at the animal facility of the Center for Molecular Biology of Inflammation (ZMBE) with routine general health checks. Work was approved by the State Agency for Nature, Environment and Consumer Protection North Rhine-Westphalia (LANUV; 84-02.05.50.15.041) and was performed in compliance with the guidelines for the welfare of experimental animals issued by the Federal Government of Germany and the state of North Rhine-Westphalia.

Golden Syrian hamsters were purchased from Janvier Laboratories, France. Eight weeks old male animals were euthanized by deep isoflurane anesthesia. After cardiac exsanguination and cervical dislocation, lung samples were collected and used for infection experiments. Ethical approval for this study was obtained from the competent authority of the Federal State of Mecklenburg-Western Pomerania, Germany upon consultation with the Ethics Committee of Mecklenburg-Western Pomerania (file number: 7221.3-1.1-041/17-5), based on national and European legislation, namely the EU council directive 2010/63/EU. Animal work at Friedrich-Loeffler-Institute was approved by the Institutional Animal Care and Use Committee (IACUC) and is continuously monitored by the Animal Welfare Officer.

### Human lung explants

Human lung tissue was obtained from patients undergoing lung surgery at the University Hospital Muenster (UKM). Written consent was given by all patients who agreed to donate their lung tissue for experimental purposes. Ethical approval was granted by the Deutsche Ärztekammer Westphalen-Lippe (AZ 2016-265-f-S). On the day of surgery, lung tissue was dissected into 100 mg tissue blocks, recovered in Roswell Park Memorial Institute Medium (RPMI) and incubated for 16 h at 37°C.

### Cells and influenza A virus strains

Madin-Darby canine kidney (MDCK) II cells and the African green monkey kidney cell line Vero E6 were propagated in Dulbecco’s modified Eagle’s medium (DMEM) containing 10% standardised fetal bovine serum (FBS Superior, Merck), 2 mM L-glutamine, 100 U/mL penicillin, 0.1 mg/mL streptomycin (1% P/S), and 1% non-essential amino acids (Merck) at 37°C and 5% CO_2_. The mouse-adapted influenza A virus strain PR8 (A/Puerto Rico/8/1934(H1N1)), the mouse-adapted highly pathogenic avian IAV variant SC35M (A/Seal/Massachusetts/1/80 (H7N7)), and the SARS-CoV-2 strain hCoV-19/Germany/NW-RKI-I-0026/2020 (β-B1.351) were provided by the strain collection of the Institute of Virology, University of Münster, Germany. IAV strains were amplified in MDCK II cells, SARS-CoV-2 was amplified in Vero E6 cells.

### Isolation of murine and hamster lungs

Murine and hamster lungs were extracted by cutting the lungs at the lung-facing site of the Bifurcatio tracheae and washed twice with PBS (D8537, Sigma Aldrich). Murine lungs weighed around 0.2 g, the weight of hamster lungs was approximately 0.8 g. To keep the ratios of virus dose per tissue weight comparable, hamster lungs were cut into smaller pieces of approximately 0.2 g. Lung tissues were placed in DMEM containing 2% BSA (Sigma).

### *Ex vivo* infection of human lung explants and drug treatment

Matching tissue samples from the same donor were utilised for the individual virus infections as published [15]. Briefly, the indicated virus dose was added to the infection medium (RPMI supplemented with 2 mM L-glutamine, 1% P/S and 0.1% BSA). Samples were placed in 1 mL virus-containing infection medium and 200 µl of the virus-containing medium were additionally injected into the tissue using a syringe. After 1 h incubation at 37°C, the medium was removed (t0) and the tissue was washed three times in PBS to remove unbound excess virus before 2 ml of fresh infection medium were added. The amount of remaining virus particles was determined in the removed virus-containing t0 infection medium and the released virus progeny was determined at 24 and 48 h post infection (hpi). For treatments, donor-matched human lung tissue was either injected for 1 h prior to viral infection with 400 µl of the infection medium containing 250 nM bafilomycin A and incubated at 37°C and 5% CO_2_ or left untreated.

### Drug treatments of murine and hamster lung tissue

Prior to viral infection, murine and hamster lung tissues were pretreated with an IFN-α subtype mixture (pbI assay science), bafilomycin A (Cayman), the Niemann-Pick inhibitor U18666A, or the antidepressant fluoxetine by injecting 400 µL of the drug-containing infection-DMEM (500 U/mL IFN-α mix, 250 nM bafilomycin A, 10µg/mL U18666A, 20 µM fluoxetine) into the tissue, followed by further incubation of the lung tissues in 1 mL of the respective drug-containing media for the indicated time at 37°C and 5% CO_2_.

### Virus infection of animal samples

Viruses were suspended in infection-PBS containing 0.2% BSA (IAV) or 2% FBS (SARS-CoV-2), 0.9 mM CaCl_2_, 1 mM MgCl_2_, 100 U/mL penicillin, 0.1 mg/mL streptomycin. For *ex vivo* infection, whole murine lungs and chopped hamster lungs were washed twice with infection-PBS and then 400 µL of the virus-containing infection-PBS were injected into the tissue using a syringe. Mock-treated lungs were injected with infection-PBS only. After an incubation period of 2 h at 37°C, tissues were washed to remove unbound viruses and were further incubated at 5% CO_2_ and 37°C. Viral titres were determined at 2, 24, and 48 hpi.

### Plaque assay

Numbers of infectious particles were determined by a standard plaque assay [19]. Briefly, MDCK II or Vero E6 monolayers grown in six-well dishes were washed with PBS and infected with serial dilutions of the respective lung homogenates in infection-PBS for 30 min at 37°C. The inoculum was then replaced with 2× MEM (MEM containing 0.2% BSA, 2 mM L-glutamine, 1 M HEPES, pH 7.2, 7.5% NaHCO_3_, 100 U/mL penicillin, 0.1 mg/mL streptomycin, and 0.4% Oxoid agar) and incubated at 37°C for 72 h. N-p-tosyl-ʟ-phenylalanine chloromethyl ketone (TPCK)-treated trypsin (Sigma-Aldrich, 1 μg/mL) was added to facilitate IAV infection in MDCK II cells. Virus plaques were visualised by staining with neutral red, and virus titres were calculated as plaque-forming units (PFU) per mL.

### Cytokine profile

Released cytokines from the *ex vivo* infected human tissues were analyzed at 24 hpi using the bead-based human Anti-Virus Response panel according to the manufacturer’s protocol (BioLegend). For the murine model, *ex vivo* infected murine lungs were flushed with 400 µL of their incubation media at 24 hpi. Cytokine profiles were analyzed using the LEGENDplex™ Mouse Anti-Virus Response Panel according to the manufacturer’s protocol (BioLegend). Bead-bound cytokines were quantified on a FACSCalibur Cytometer (Becton Dickinson). Concentrations were calculated using the LEGENDplex™ Data Analysis Software (BioLegend).

### RNA extraction from lung tissues and real-time quantitative PCR

Murine tissues were homogenised in peqGOLD TriFast™ (VWR Peqlab), using lysing matrix tubes (MD Biomedicals). Total RNA was isolated according to the manufacturer’s instructions, and 1 µg was converted into cDNA using the High-capacity cDNA reverse transcription kit and random primers (Applied Biosystems). Lung gene expression profiles were analyzed either by SYBR Green qPCR (Platinum® SYBR® Green qPCR SuperMix-UDG w/ROX, Thermo Fisher Scientific) for M1 of IAV (Microsynth custom-made primers, vM1_fwd 5’-TGCAAAAACATCTTCAAGTCTCTG-3’, vM1_rev 5’-AGATGAGTCTTCTAACCGAGGTCG-3’) or by TaqMan primer/probe sets (IFN-α, IFN-β, IFN-γ, IL-6, CCL2, Mx1, DDX58 (RIG-I)) from the Universal ProbeLibrary (Roche). Glyceraldehyde-3-phosphate dehydrogenase (GAPDH) and cytochrome c (CYCS) were used as internal references. Human lung tissue samples were stored in RNAlater (Sigma, Germany) at −80°C until disruption in RLT buffer supplemented with 1% β-Mercaptoethanol (Sigma, Germany), using Lysing Matrix A tubes and the FastPrep-24 disruptor (MP Biomedicals, Germany). Total RNA was purified using the RNeasy Plus mini kit (Qiagen, Germany) according to the manufacturer’s instructions and transcribed using oligo (dT) primers and revertAid H Minus Reverse Transcriptase (Thermo Fisher Scientific, USA). Primers specific to the indicated genes were used to quantify mRNA expression. Samples from five independent experiments were run in triplicates on a LightCycler® 480 Instrument II (Roche). Changes in gene expression were calculated by the delta-delta-Ct method [20]. Statistical significance of the differences was evaluated by Mann–Whitney U-tests on the ΔΔCt values.

### Lung histology

Isolated lung tissues were fixed in 4% formaldehyde solution (pH 7.4) for 4 h, then dehydrated with a graded series of isopropanol (70% – 100%) and embedded in paraffin. Tissue sections (4 µm) were produced using the microtome 355S (Thermo Scientific). After rehydration in 10 mM citric acid buffer (pH 6.0, Roth) for 20 min and heat-mediated antigen retrieval, the paraffin sections were blocked in PBS containing 10% FBS and 0.1% Triton-X-100 for 30 min. Influenza virus nucleoprotein (NP) was stained with anti-NP antibody (goat anti-influenza NP [G105] 1:2.500, kind gift by Dr. Robert Webster), murine prosurfactant protein-C (proSP-C) was stained with anti-proSP-C antibody (rabbit anti-proSP-C 1:2.000, Millipore, AB3786), and hematoxylin and eosin (H&E) staining (Roth) was performed according to the manufactureŕs protocol. Tissue sections were incubated with the appropriate primary antibodies at RT for 1 h, followed by incubation for 30 min with the species-specific biotinylated secondary antibodies (Goat anti-rabbit IgG (H + L) (ab64256), goat anti-mouse IgG (H + L) (ab64255)). The Vectastain ABC-AP Kit (Vector Laboratories, (Cat. No.: AK-5000)) was used for visualisation of the stained proteins as described in the manufactureŕs protocol. Images were obtained on a KEYENCE BZ-9000 microscope. For each condition, three different sections per mouse lung (approx. 250 µm apart from each other) were quantified from at least five mice. The area of the NP-positive foci was measured and analyzed for total nuclei section areas of the specimen. To assess the tissue integrity, hematoxylin and eosin-stained 4 μm-sections were analyzed for tissue densification as an indicator of immune cell accumulation and fibrosis. All analyses were quantified in a blinded manner via the Keyence BZ Analyzer (Keyence) software.

### Data and statistical analysis

A priori power analysis using G*Power 3.1 was performed to determine the sample sizes required to detect > 90% reduction in virus titres at powers > 0.8.

Data on infectious viral titres, cytokine profiles, gene expression, and histological staining of viral NP, ProSP-C, and H&E are presented as means ± SEM of at least five per condition. Differences between conditions were analyzed for statistical significance by Mann–Whitney U-test or by Kruskal–Wallis test as indicated, followed by Dunn's multiple comparisons post-test. Data were analyzed using the GraphPad Prism version 8.00 software (GraphPad). Curve slopes were calculated using the in-built model “non-linear fit with exponential growth with log(population)” and expressed as means ± SEM.

## Results

### Influenza A virus infection of murine and human lung tissue models resulted in similar progeny virus titres

**Figure 1.**
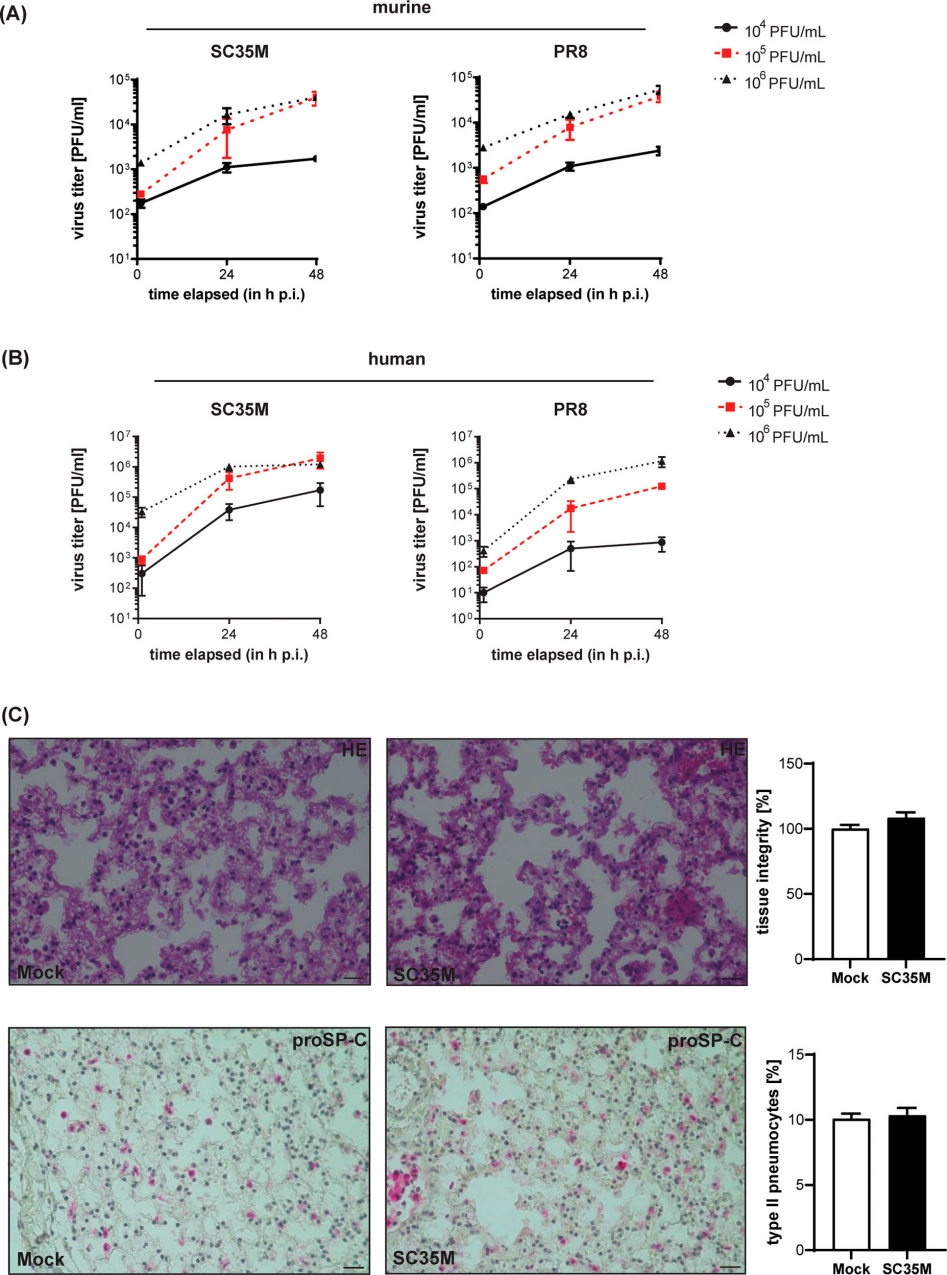
Ex vivo infection of murine and human lung tissues. (A, B) Kinetics of influenza PR8 and SC35M replication. Extracted (A) mice lungs and (B) human lung explants were infected with the indicated viral doses (PFU/mL) for 2, 24 and 48 h. Viral titres were determined via plaque assay. (C) Representative images of histopathologic condition (H&E stain, upper panel) and distribution of proSP-C positve cells (lower panel) of murine lung tissue sections at 24 hpi. n = 5 murine lungs/condition, scale bar 50 µm. To score lung inflammation, separate sections of each lung were evaluated for tissue density, and the mean density/lung was calculated, normalised to mock (100%) and compared by Mann-Whitney U test. Numbers of type II pneumocytes were counted, normalised to the numbers of nuclei detected in the corresponding tissue section, and analyzed by Mann-Whitney U test.

To determine the dose that is required for reliable experimental *ex vivo* infections, we assessed viral titres in the lungs 24, and 48 h post-infection (hpi) with the indicated viral doses. Titres obtained at 2 hpi served as an input control to determine the initial viral load in the lung tissue. As shown in Figure 1A, progressive viral replication ≥ 1 log10 difference was observed in both human and murine tissues at 48 hpi. Infection with an initial viral dose of 10^5^ PFU/mL resulted in the strongest increase in viral titres for both IAV viruses and murine and human lungs as revealed by curve slope calculations (for SC35M-infected murine lungs: 33.08 ± 5.34 for 10^4^ PFU/mL, 853 ± 264.7 for 10^5^ PFU/mL, and 838.2 ± 119 for 10^6^ PFU/mL; for PR8-infected murine lungs: 39.74 ± 40.71 for 10^4^ PFU/mL, 944.1 ± 237.7 for 10^5^ PFU/mL, and 864.6 ± 133 for 10^6^ PFU/mL; for SC35M-infected human lungs: 3653 ± 2025 for 10^4^ PFU/mL, 41056 ± 18314 for 10^5^ PFU/mL, 24976 ± 6786 for 10^6^ PFU/mL; for PR8-infected human lungs: 18,21 ± 10.57 for 10^4^ PFU/mL, 2657 ± 669.2 for 10^5^ PFU/mL, and 24559 ± 8413 for 10^6^ PFU/mL), and was therefore used for subsequent infection experiments (Figure 1AB). We also analyzed the murine tissues at 24 and 48 hpi via hematoxylin–eosin staining to assess tissue integrity and also stained for proSP-C, a specific marker for type II pneumocytes. In both mock-treated and SC35M-infected lung tissues, a similar distribution of type II pneumocytes and comparable tissue integrities were observed (Figure 1C, supplement Figure 1). Both the isolated human and murine lung tissues, support productive IAV replication for 48 hpi at least for the mouse-adaptive IAV strains SC35M and PR8 without affecting tissue integrity or the abundance of type II pneumocytes.

### Gene expression profiles and cytokine levels of *ex vivo* infected murine and human tissues

**Figure 2.**
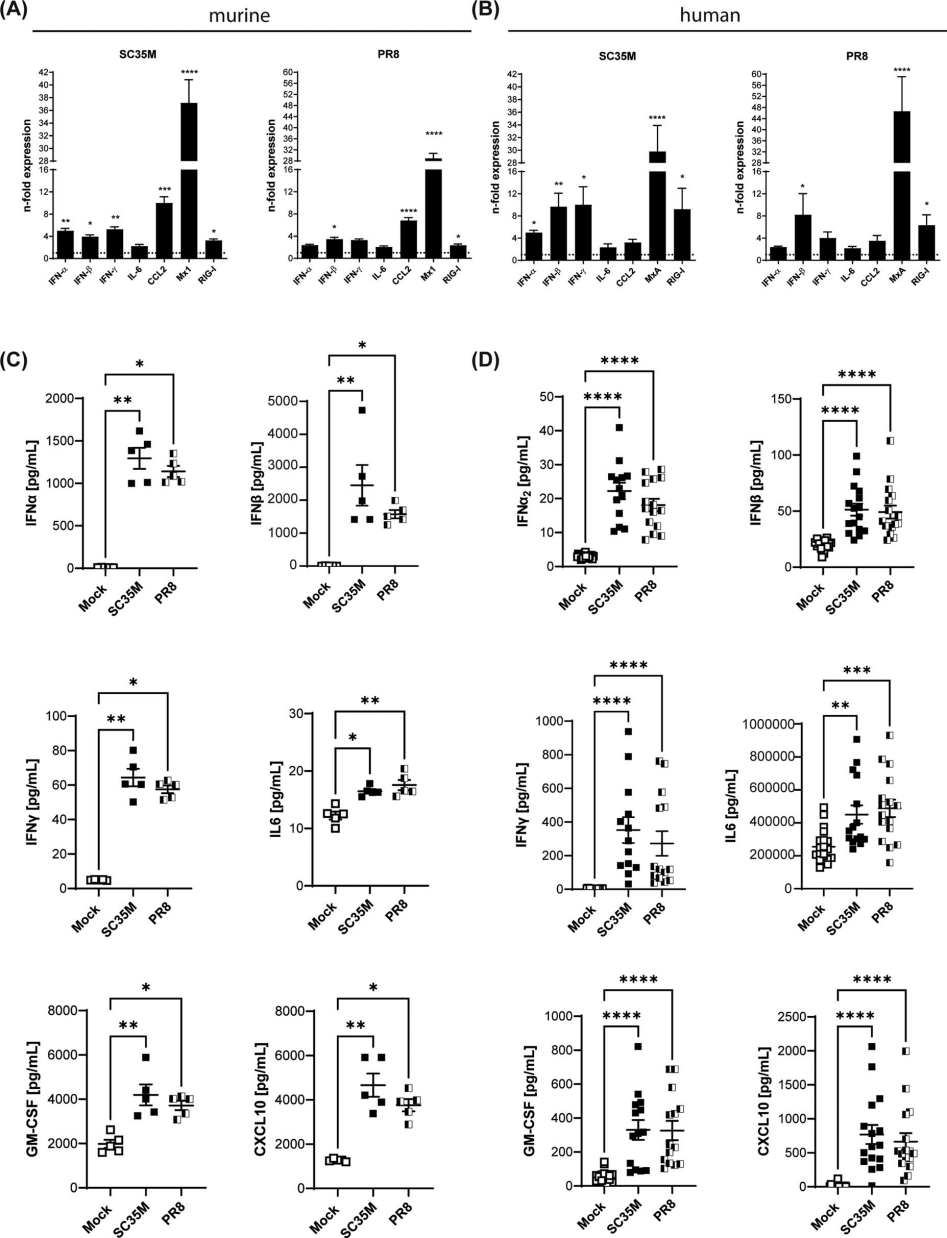
Antiviral response to SC35M and PR8 infection. Gene expression of IFNs and ISGs in SC35M- or PR8-infected (A) murine and (B) human lung tissues (10^5^ PFU/mL, 48 hpi) were analyzed by qPCR and normalised to mock-infected samples. Bars represent fold change of relative expression levels ± SEM, dotted lines indicate no change in gene expression. Cytokine profile of infected (C) murine and (D) human lung tissues at 24 hpi. Scatter plots of individual murine lung tissue data with means ± SEM. N = 5 murine lungs/group, Kruskal Wallis followed by a Dunn’s post-test, * *p* < 0.1, ** *p* < 0.01, *** *p* < 0.001, **** *p* < 0.0001.

We next characterised the host innate immune response to the IAV strains PR8 and SC35M in the *ex vivo* infected tissues at 48 hpi by gene expression analysis for several antiviral genes and cytokines in total lung lysates. As expected, IAV infection induced the antiviral interferon response in both the murine and human lung tissues, as confirmed by the significantly enhanced expression of interferons and the interferon-stimulated genes (ISGs) Myxoma Resistance Protein 1/A (Mx1/MxA) and retinoic acid-inducible gene I (Rig-I) (Figure 2AB, suppl. Figure 2A). In the murine tissues, the expression level of the anti-inflammatory cytokine CCL2 was also increased (suppl. Figure 2B). We also evaluated the secretion levels of several cytokines and chemokines by a bead-based multiplex assay. In line with the data obtained from gene expression analysis at 48 hpi, SC35M- and PR8-infected lung tissues at 24 hpi released significantly enhanced amounts of the type I IFN subtypes IFN-α2 and IFN-β, IFN type II (IFN-γ), GM-CSF, CXCL10, and IL-6 (Figure 2CD). Interestingly, murine lungs responded to infection with a much stronger release of type I interferons than the human lungs. In contrast, the type II interferon response was much stronger in the human tissue. The secretion of the different IFNs suggested the involvement not only of epithelial (type I IFNs) but also of immune cells (type II IFNs) in the antiviral response to SC35M IAV infection in *ex viv*o-infected lung tissues. We also observed at least in the human *ex vivo* model enhanced secretion of IL-1β, TNF-α, IL12p70, IFN-λ, and IL-10, while Il-1β, TNF-α, and IL-12p70 were not significantly altered in the murine model (suppl. Figure 2BC).

### The endolysosomal host–pathogen interface as a pharmacological target

**Figure 3.**
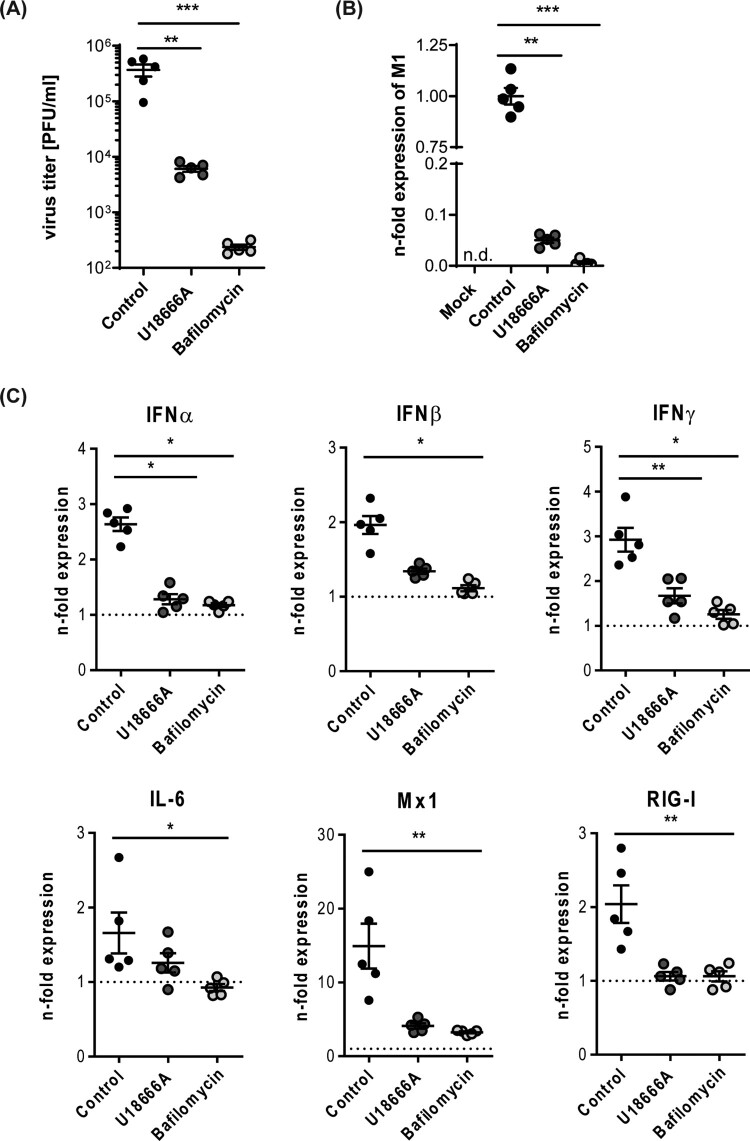
Impact of compromised endolysosomes on SC35M infection. Murine lung tissues were either treated with 250 nM bafilomycin A for 1 h or with 10 µg/mL U18666A for 16 h prior to *ex vivo* infection with the IAV strain SC25M for 24 h. Control, no pre-treatment, infection; Mock, no pre-treatment, no infection (A) Viral titres detected in lung homogenates (PFU/mL). (B) Expression levels of viral M1 gene in non-treated compared to pre-treated SC35M-infected lungs. Data are expressed as fold changes ± SEM compared to control, the cut-off for detected signal was set to Ct ≤ 35. (C) Scatter plot representation of host cell gene expression levels in SC35M-infected lungs compared to mock (pointed lines). Data of individual mouse lungs are expressed as fold changes with means ± SEM superimposed. Statistical significance of the differences was evaluated by Kruskal-Wallis test on ΔΔCt values followed by Dunn's multiple comparisons test. Data were analyzed by Kruskal-Wallis test followed by a Dunn's multiple comparisons test, *p < 0.05, **p < 0.01, ***p < 0.001, n.d.    not detectable.

The similar kinetics and comparable antiviral responses upon *ex vivo* infection with the two different IAV strains suggested that our murine lung infection model faithfully reproduced the early IAV infection phases seen in the human tissue. To assess whether pharmacological interventions might also be addressed in the tissue explant model, we focused on treatment regimens known to block IAV infection in cell culture models effectively. Thus, we pre-treated lungs for 16 h with the macrolide antibiotic bafilomycin A, an inhibitor of vacuolar-type H^+^-pumps that prevents the endosomal acidification that is essentially required for viral endosomal escape, and the Niemann-Pick C1 (NPC1) inhibitor U18666A which causes an antiviral cholesterol accumulation in endolysosomes. Notably, both drugs that severely inhibit IAV endosomal escape *in vitro* also significantly reduced viral titres and viral gene expression in the murine lung tissue (Figure 3AB, and suppl. Figure 3), thus confirming that the well-documented antiviral effects could be faithfully replicated in our *ex vivo* model. Analysis of the cytokine and antiviral protein response (Figure 3C) also endorsed the lower infection levels in pre-treated lungs.

**Figure 4.**
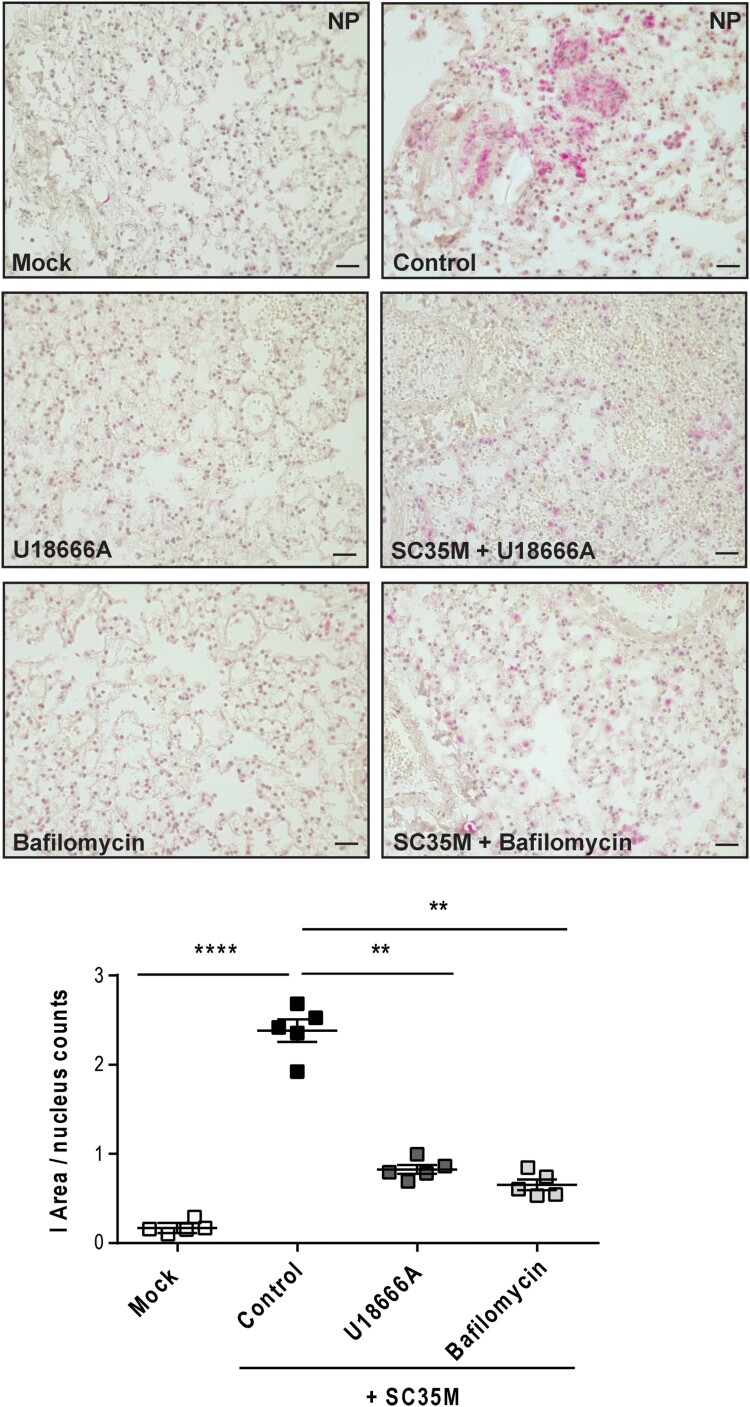
Histological evaluation of the IAV infection rate. Murine lungs were either pre-treated with the solvent DMSO (control), 250 nM bafilomycin A (1 h) or with 10 µg/mL U18666A (16 h) prior to infection, followed by an infection with 10^5^ PFU/ mL of IAV strain SC35M for 24 h. Representative images of NP immunostaining detected in the lung sections. Quantitative analysis of NP staining. Scatter plot representation of individual lungs, with means ± SEM superimposed. Data were analyzed by Kruskal-Wallis test followed by Dunn's multiple comparisons test, * *p* < 0.1, ** *p* < 0.01, **** *p* < 0.0001, n = 5 murine lungs/group, scale bar 50 µm.

Next, we evaluated the efficacy of both drugs to reduce the numbers of infected cells by histopathology. Thus, we pre-treated murine lung tissues either with 250 nM bafilomycin A for 1 h or with U18666A for 16 h before infection with 10^5^ PFU/ mL of the IAV strain SC35M for 24 h. We detected significantly reduced numbers of NP-positive, i.e. infected cells in lungs that were pre-treated with bafilomycin A or U18666A (Figure 4), thus correlating the previously observed reduction in viral titres with the levels of NP-positive cells and the significantly decreased IAV M1 gene expression levels (Figure 3 and 4).

### Assessing the antiviral capacity of the host-targeting drugs IFN-α and fluoxetine

**Figure 5.**
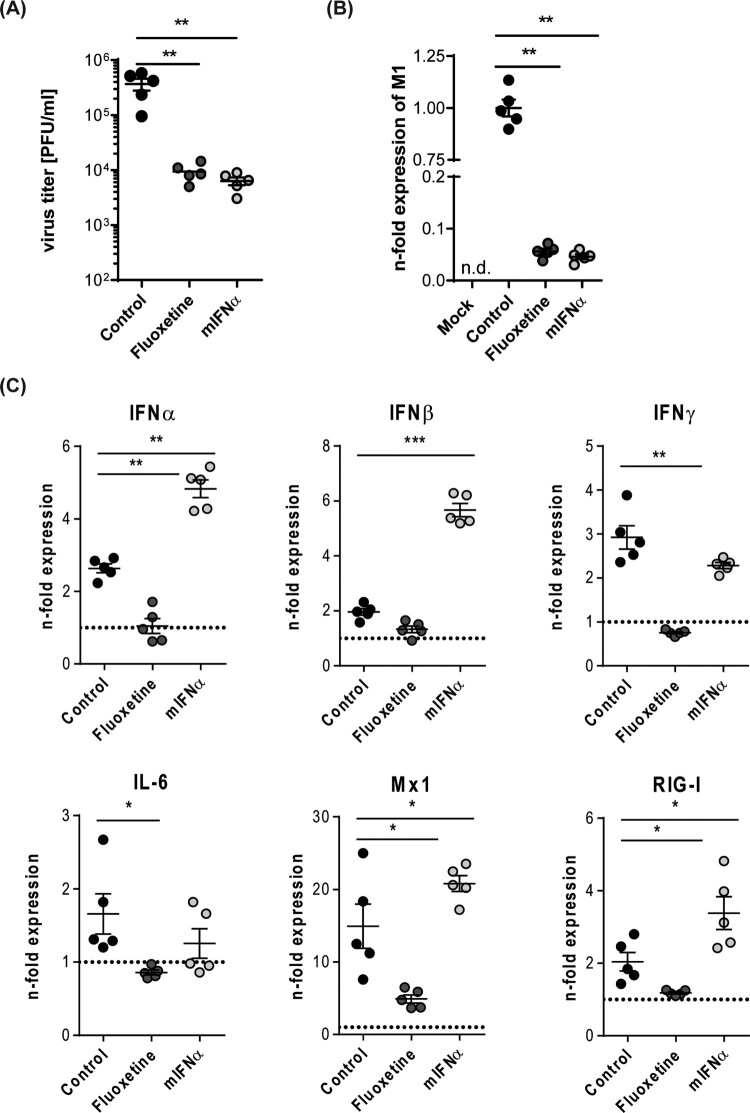
Treatment with mIFN-α or the repurposed FIASMA fluoxetine impaired viral infection. Murine lung tissues were either pretreated for 8 h with the solvent, 20 µM fluoxetine, or with 500 U/mL IFN-α. (A) Virus titres determined in murine lungs infected with 10^5^ PFU/mL SC35M for 24 h. (B, C) Viral and antiviral gene expression were analyzed to assess the antiviral response in non-treated and treated SC35M-infected lungs. Data were expressed as relative expression levels ± SEM relative to the two reference genes GAPDH and ACTB. Symbols represent values of individual mouse lungs, with the means ± SEM superimposed. Data were analyzed by Kruskal-Wallis test followed by a Dunn's multiple comparisons test, *p < 0.05, **p < 0.01, ***p < 0.001, n.d.    not detected.

**Figure 6.**
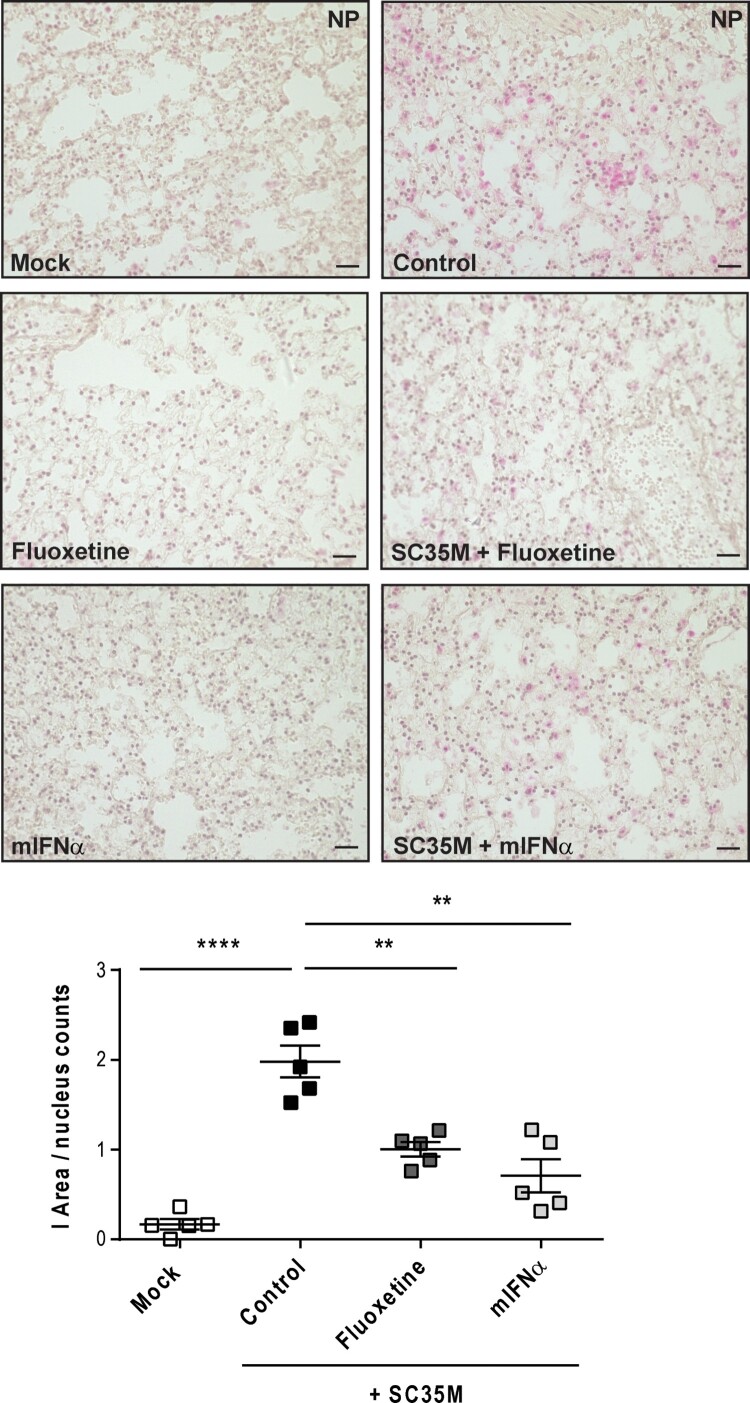
Histological evaluation of SC35M infection rate. Murine lungs were either pre-treated with the solvent DMSO (control), 20 µM fluoxetine (8 h), or with 500 U/mL IFN-α (8 h) prior to infection with 10^5^ PFU/ mL SC35M for 24 h. Representative images of lung tissue sections stained for viral NP. Scatter plot representation of the total area covered by NP-positive cells. Data showing single lungs, with means ± SEM superimposed. Statistically significant differences were detected by Kruskal-Wallis test and Dunn's multiple comparisons test, * *p* < 0.1, ** *p* < 0.01, **** *p* < 0.0001, n = 5 murine lungs/group, scale bar 50 µm.

Although both U18666A and bafilomycin A have antiviral activity in cell culture infection models, both agents are not approved for clinical use due to their unfavourable safety profiles [21–24]. To explore the suitability of our *ex vivo* mouse tissue model for drug assessment studies, we next included drugs that were already suggested to have repurposing potential for antiviral approaches [25]. The widely used antidepressant fluoxetine was already shown to inhibit IAV and SARS-CoV-2 infection *in vitro* [19,26], most likely based on its chemical nature as a functional inhibitor of the acid sphingomyelinase (FIASMA) in endolysosomes [27]. IFN-α induces the onset of the antiviral response and the expression of antiviral restriction factors including Mx1 and RIG-I [28]. Of note, we observed for both drug treatments a significant reduction in viral titres (Figure 5A) which was reflected in the lower expression of the viral protein M1 in the drug-treated samples (Figure 5B). We further examined the expression of cytokines and ISGs that are pivotal for the shaping of the cellular and innate immunity upon IAV infection. In fluoxetine-treated samples, the expression levels of IFNs, IL-6, and the ISGs Mx1 and RIG-I were markedly lower than in the control samples. Treatment with IFN-α elevated the expression levels of IFN-α, IFN-β, Mx1 and RIG-I. IFN-γ and IL-6 levels were not significantly affected (Figure 5C). To monitor whether the viral titres and the inflammatory stage of the tissues correlated with the number of infected cells, we performed a viral NP staining and quantified the infected area (I-area) based on the proportion of NP-positive cells. As expected, we observed for both drug treatments decreased levels of infected cells which matched with the lower viral titres upon treatment (Figure 6).

### Suitability of mouse and hamster *ex vivo* infection models for studying SARS-coV-2 infection

**Figure 7.**
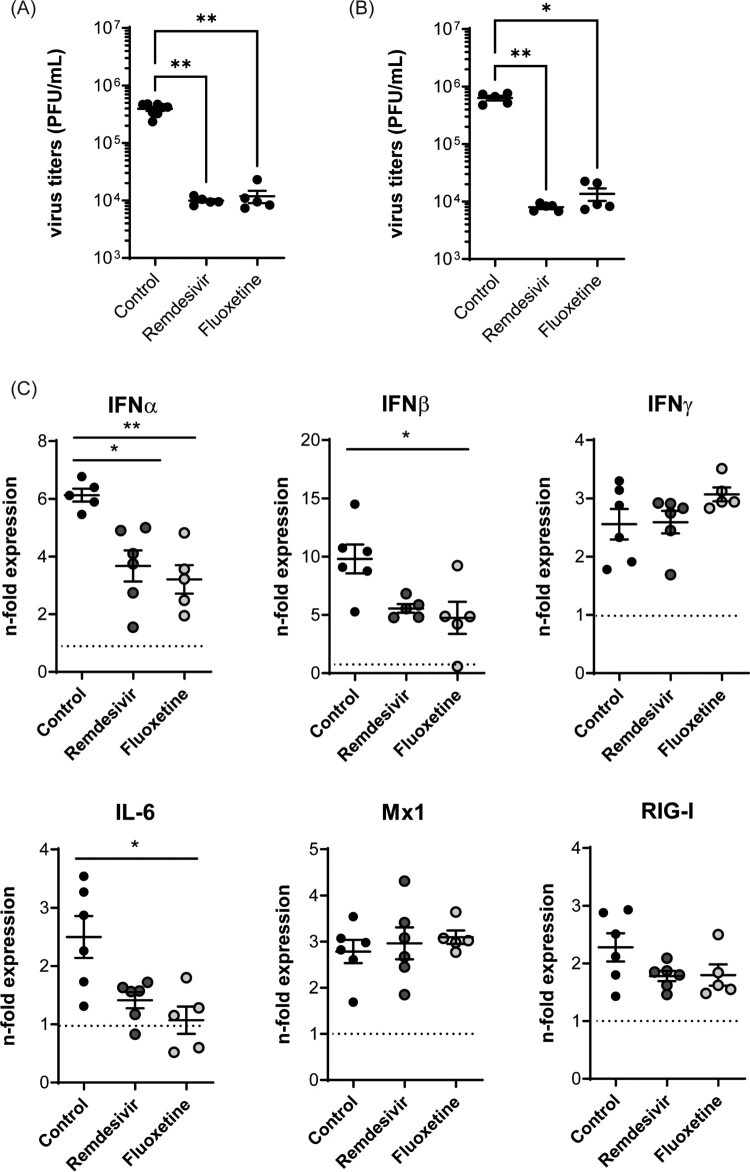
SARS-CoV-2 infections of (A) hamster and (B) murine lung explants. Lungs were infected with 10^5^ PFU/mL of the SARS-CoV-2 variant B1.531 for 72 h. Post-infection treatment with remdesivir [2 µM], or fluoxetine [20 µM] was initiated 2 hpi. Viral titres are expressed as PFU/per mL detected in the individual lung samples, with means ± SEM superimposed. (C) Antiviral gene expression was analyzed to assess the antiviral response in non-treated and treated SARS-CoV-2-infected murine lungs. Data were expressed as relative expression levels ± SEM relative to the two reference genes GAPDH and actin β. Symbols represent values of individual mice lungs, with the means ± SEM superimposed. Data were analyzed by Kruskal-Wallis test followed by a Dunn's multiple comparisons test, *p < 0.05, **p < 0.01, ***p < 0.001, n.d.    non-detectable; n ≥ 5 murine lungs/group.

The ongoing SARS-CoV-2 pandemic and associated COVID-19 has drastically shown the urgent need for the rapid development of pharmaceutical approaches to deal with emerging viruses. While only a few of the identified SARS-CoV-2 variants such as Beta B1.351 do replicate in mice [29], the Syrian hamster is a natural host and therefore serves as an experimental small animal model to study SARS-CoV-2 infection [13], To assess whether animal work can also be replaced in this host, we evaluated the suitability of isolated hamster lungs for studying SARS-CoV-2 infection *ex vivo* with the Beta variant B1.351, which allowed us to also investigate *ex vivo* infection in the murine lung tissues. As shown in Figure 7, we detected SARS-CoV-2 propagation in both the hamster and the murine lung tissue samples 72 hpi. Notably, the titres obtained from *ex vivo* infected hamster lungs (Figure 7A) were comparable to those observed in the murine lung explants (Figure 7B). Similar to what was observed earlier for SARS-CoV-2 infection using permissive human lung cell lines [19,26] and human lung tissue slides [30,31], both fluoxetine and the direct antiviral remdesivir reduced viral titres even in the post-infection treatment in both species (Figure 7A, B).

## Discussion

Here, we established an easy-to-use murine *ex vivo* infection model based on lung tissue explants which was used to analyze viral replication kinetics and antiviral responses of the mouse adapted IAV strains PR8 and SC35M, and the SARS-CoV-2 variant B.1.351. We aimed to develop an experimental model that recapitulates the initial phase of viral airway infection in a physiologically meaningful setting that can be set up in a standard cell culture lab at the required safety level without sophisticated equipment and experimentation skills or animal experimentation. The ability of the murine lung tissue explants to support productive replication of the mouse-adapted IAV strains and the only known SARS-CoV-2 strain (β-B1.351 variant) that has been reported to infect wild-type mice, similar to the kinetics observed in lung explants from the respective natural host, i.e. human and hamster, confirms their usability as a robust infection model. Analysis of the innate immune responses in murine in human lungs revealed similarities and differences upon viral infection. Increased expression levels of the type I interferons IFN-α, IFN-β, and the type II interferon IFN-γ indicated the onset of the interferon response, a well-known antimicrobial programme to induce cellular antimicrobial and particularly antiviral defense, that is activated upon sensing of microbial products [32]. Accordingly, we also detected elevated gene expression levels of the dynamin-like GTPase Mx1/MxA which has antiviral activity against a wide range of mostly RNA viruses, and the cytosolic pattern recognition receptor RIG-I that recognises short viral double-stranded RNA. Both proteins are interferon-stimulated genes (ISGs) [33,34], thus pointing to the induction of the canonical type I interferon signalling pathway. Our analysis revealed the elevated expression of the pro-inflammatory cytokine IL-6 in the murine lungs which has been shown to positively correlate with disease progression and respiratory failure (Santa Cruz 2021). In this regard, the dysbalanced cytokine response (“cytokine storm”) and the concomitant hyper-inflammation are the major cause of disease severity and fatal outcome of HPAIV and SARS-coV-2 infection. The secretome analysis confirmed an IFN-induced antiviral response, as we found elevated secretion of the interferons IFN-α, IFN-β, as well as cytokines CCL2 and CCL5, both of which are produced upon type I interferon induction and are involved in the recruitment of immune cells, and the IFN-γ-induced pro-inflammatory chemokine CXCL10. Excessive recruitment of immune cells and inflammatory remodelling of the tissue cytoarchitecture transitions the tissue to fibrosis. Of note, we also detected increased secretion of the immune mediator GM-CSF, which is lung-protective during influenza virus infection and improves epithelial repair [35]. Taken together, our data suggest that the murine *ex vivo* model is faithfully recapitulating the host innate immune response profile involved in the generation of an antiviral cellular environment.

To confirm the suitability of the model for studying the virus-host interplay during viral infection, we treated lung tissues with the macrolide antibiotic bafilomycin A1 or the NPC1 inhibitor U18666A. Both agents target the endolysosomes [36,37], the sites of actual IAV entry into the host cell [38]. Bafilomycin A1 specifically inhibits the endolysosomal proton pump vacuolar-type H^+^-ATPase (V-ATPase) which acidifies the lumen [39] U18666 targets the endolysosomal cholesterol exporter NPC1, resulting in cholesterol-laden endolysosomes. Both these endolysosomal manipulations interfere with virus-endosome fusion [36,40] *in vitro*. The marked reduction in IAV titres and numbers of infected cells that correlated with a reduced immune response confirm that the endolysosomal host–pathogen interface can be targeted *ex vivo*, with the lower virus titres leading to a weaker inflammatory response.

Because their unfavourable safety profiles limit the clinical use of U18666A and bafilomycin A1 [21–24], several studies have already explored the endolysosomal virus-host interface as a potential target for antiviral drugs utilising drug repurposing strategies [36,41–45]. To assess whether our infection model is also suited for the evaluation of pharmacological interventions, we included treatment of the tissues with the clinically licensed antidepressant fluoxetine that has been reported to interfere with early steps in IAV and SARS-CoV-2 infection, probably at the stage of endosomal escape [46,47]. Indeed, viral titres and numbers of infected cells were reduced, and the interferon response was weaker. In this regard, U18666A, bafilomycin A, and fluoxetine were quite comparable, in line with their comparable modes of action, i.e. lowering the initial viral entry by interfering with endosomal escape of the enveloped viruses. We also tested IFN treatment to assess infection of an already established antiviral environment. As expected, type I IFN treatment enhanced the expression of the antiviral restriction factor Mx1/MxA and the RNA receptor RIG-I and resulted in reduced viral replication, supporting the antiviral potential of IFN treatment.

Finally, we also explored whether the presented murine *ex vivo* model can be used to investigate SARS-CoV-2 infection. The results seen in murine or hamster lung tissues treated with fluoxetine or the direct antiviral remdesivir are in line with earlier reports. Interestingly, both drugs reduced the virus-induced expression of type I IFN and the prognostic factor IL-6, while the expression of the type II IFN and of the ISGs Mx1 and RIG-I was not altered upon treatment, indicating that the reduced IFN levels were still sufficient enough to establish an antiviral response in the tissue.

Taken together, our data suggest that the mouse *ex vivo* lung model is suited as a preclinical model to recapitulate the innate immune response to viral infection and can be used as a convenient model for drug testing. Moreover, the hamster tissue explant model might be employed to study virus-host interactions and host responses upon infection with SARS-CoV-2 subtypes that do not replicate in mice.

## Supplementary Material

Supplemental Material

## Data Availability

The data that support the findings of this study are available from the corresponding author upon reasonable request. Some data may not be made available because of privacy or ethical restrictions.
