# Supplementary material for: 3D Ex vivo tissue platforms to investigate the early phases of influenza a virus- and SARS-CoV-2-induced respiratory diseases
Source: Emerg Microbes Infect. 2022 Sep 21;11(1):2160–75. doi: 10.1080/22221751.2022.2117101 (PMC9518268; doi:10.1080/22221751.2022.2117101)
Supplement: Supplemental Material [file TEMI_A_2117101_SM8340.docx]

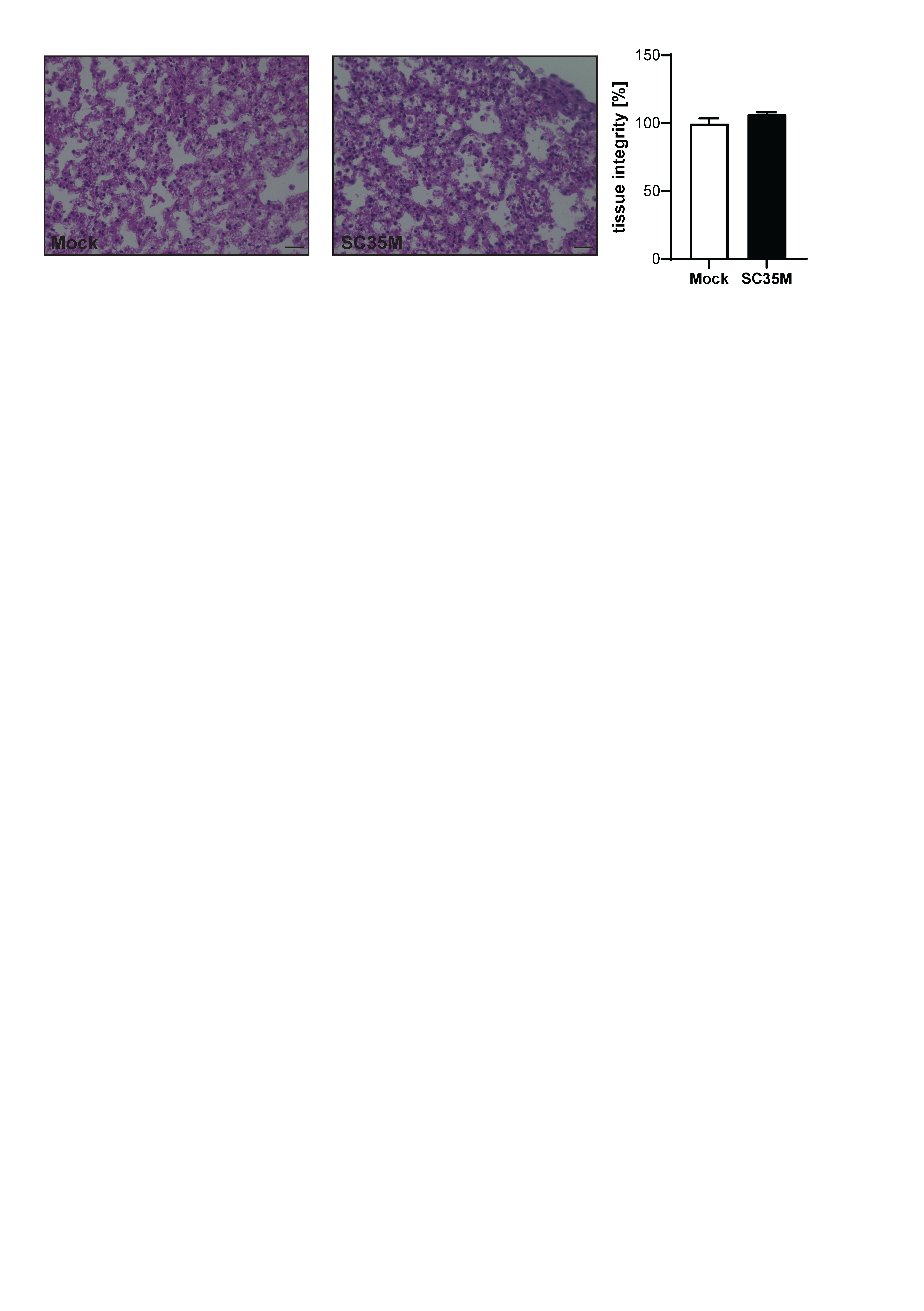


**Suppl. Figure 1.** Histological examination of the tissue integrity of Mock or SC35M-infected murine lungs 48 hpi. Data are expressed as mean ± SEM, n = 5 murine lungs/group, scale bar 50 µm.


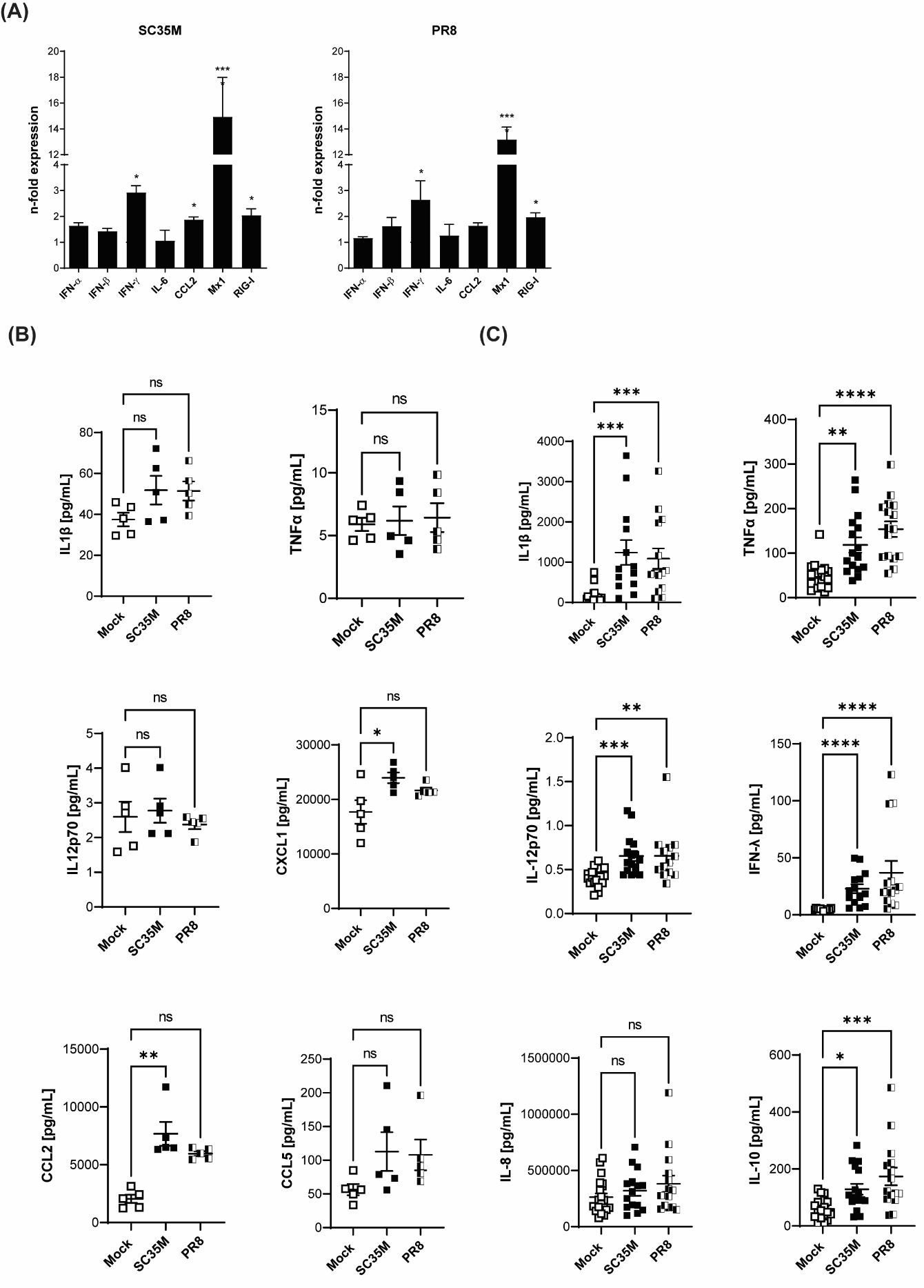


**Suppl. Figure 2.** Inflammatory response in SC35M- and PR8-infected murine lung tissues. Expression of the indicated genes were analyzed upon infection 24 hpi. (B, C) Analysis of the cytokine release in SC35M-infected (B) murine and (C) human lung tissues. Lungs were either infected with SC35M using 10^5^ PFU/mL for 2 h and cytokine levels measured by a bead-based multiplex assay. Data are expressed as scatter plots of individual murine lung tissue data with the means ± SEM, n = 5 murine lungs/group. Data were analyzed by Kruskal Wallis followed by a Dunn’s post-test, *p < 0.05, **p < 0.01, ***p < 0.001, ****p < 0.0001.


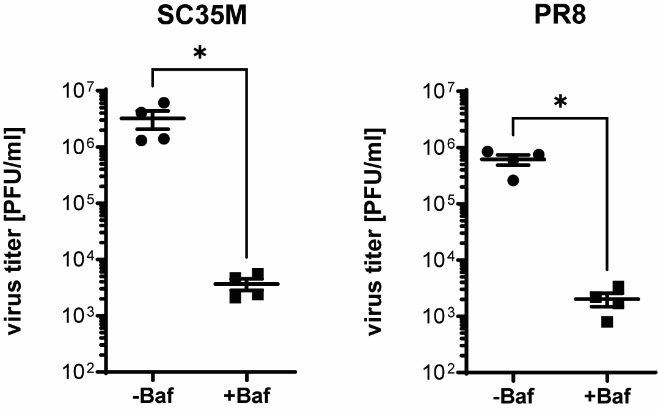


**Suppl. Figure 3.** Bafilomycin treatment in human lung explants. Lungs were infected with 10^5^ PFU/mL for 2 h, followed by a treatment with the solvent DMSO or bafilomycin and viral titers were taken upon 24 hpi. Viral titers are expressed as PFU/mL and each scatter dot represents an individual sample. Data were analyzed by Mann Whitney test, *p < 0.05.
